# Effective Estimation of Dynamic Metabolic Fluxes Using ^13^C Labeling and Piecewise Affine Approximation: From Theory to Practical Applicability

**DOI:** 10.3390/metabo5040697

**Published:** 2015-12-04

**Authors:** Robin Schumacher, S. Aljoscha Wahl

**Affiliations:** Department of Biotechnology, Delft University of Technology, Julianalaan 67, 2628 BC Delft, The Netherlands; E-Mail: s.a.wahl@tudelft.nl

**Keywords:** hybrid system, dynamic metabolic flux analysis, DMFA, ^13^C, shape-prescriptive modeling, implicit filtering, fluxomics

## Abstract

The design of microbial production processes relies on rational choices for metabolic engineering of the production host and the process conditions. These require a systematic and quantitative understanding of cellular regulation. Therefore, a novel method for dynamic flux identification using quantitative metabolomics and ^13^C labeling to identify piecewise-affine (PWA) flux functions has been described recently. Obtaining flux estimates nevertheless still required frequent manual reinitalization to obtain a good reproduction of the experimental data and, moreover, did not optimize on all observables simultaneously (metabolites and isotopomer concentrations). In our contribution we focus on measures to achieve faster and robust dynamic flux estimation which leads to a high dimensional parameter estimation problem. Specifically, we address the following challenges within the PWA problem formulation: (1) Fast selection of sufficient domains for the PWA flux functions, (2) Control of over-fitting in the concentration space using shape-prescriptive modeling and (3) robust and efficient implementation of the parameter estimation using the hybrid implicit filtering algorithm. With the improvements we significantly speed up the convergence by efficiently exploiting that the optimization problem is partly linear. This allows application to larger-scale metabolic networks and demonstrates that the proposed approach is not purely theoretical, but also applicable in practice.

## 1. Introduction

In the natural environment, microorganisms are exposed to transient environmental conditions that can trigger a wide range of genetic, regulatory and metabolic responses to adapt and survive [1,2]. These adaption mechanisms are described to operate at very different timescales:

(1) Genetic adaption by mutagenesis and selection with a timescale of several generation times [3],
(2) Adaption of gene expression levels with a timescale of minutes [4],
(3) Post-translational modifications with a time constant on the order of seconds and
(4) Kinetic response which is considered an inherent property of the enzymes in the metabolic network and therefore persistent.

Kinetic metabolic regulation is a result of complex interactions between enzymes, substrates and allosteric effectors. Enzyme kinetics can be investigated *in vitro*, but the validity of estimated kinetic parameters has been shown to be limited for reconstruction of the observed *in vivo* metabolic network properties [5]. *In vivo*, only the whole network response can be analyzed, making the identification of kinetics highly challenging. Pioneering *in vivo* research [6] used stimulus-response experiments, where typically a perturbation is introduced to the extracellular space and the (intracellular) response of the system is captured by rapid sampling and quantitative metabolomics. The observations are then interpreted by generation of kinetic model(s) and parameter fitting.

These models use kinetic formalisms to describe the flux as a function of enzyme activity (*e*), substrate and effector concentrations (**c**_i_) and kinetic parameters (*θ*) and are typically non-linear. The latter can be either derived from mechanistic assumptions e.g., Michaelis-Menten kinetics, or be non-mechanistic e.g., power law leading to parameters with no physical meaning [7].

(1) $$v=e\cdot f(c_{i},\theta )$$

There are mainly two inherent conceptual drawbacks of kinetic modeling:

(1) The model equations (metabolite balances) are strongly dependent on each other, which means the system can hardly be solved from a decomposition and commonly also leads to highly correlated parameters [8,9].
(2) The kinetic functions have to be chosen *a priori*, which means that the mechanism of every enzyme and all interactions between metabolites and enzymes involved in the regarded metabolic network have to be selected before parameter optimization. Especially, the kinetic formats of each reaction in larger metabolic networks are yet unknown, unclear, or shall be deduced from the captured observables.

One solution that has been used to address this issue was to define a family of different models, also referred to as ensemble modeling [10,11,12,13], where it is good practise to find the simplest model that can describe the data (Occam’s razor). Also, strategies to speed up parameter estimation in kinetic models have been proposed using the estimation of fluxes from the metabolic network stoichiometry as an intermediate step and scaffold [14,15]. This approach allows exploiting the linear properties of the stoichiometry matrix, *i.e.*, the nullspace, however, the methodology requires selecting a kinetic model *a priori* and cannot directly identify dynamic metabolic fluxes. Regardless of those challenges, the number of uncertain reaction mechanisms in a kinetic model leads to a rapidly increasing number of models to be compared, together with the challenges of parameter estimation in non-linear systems, which result in putative non-convex optimization landscapes that quickly reach computationally infeasible scales.

**Figure 1:**
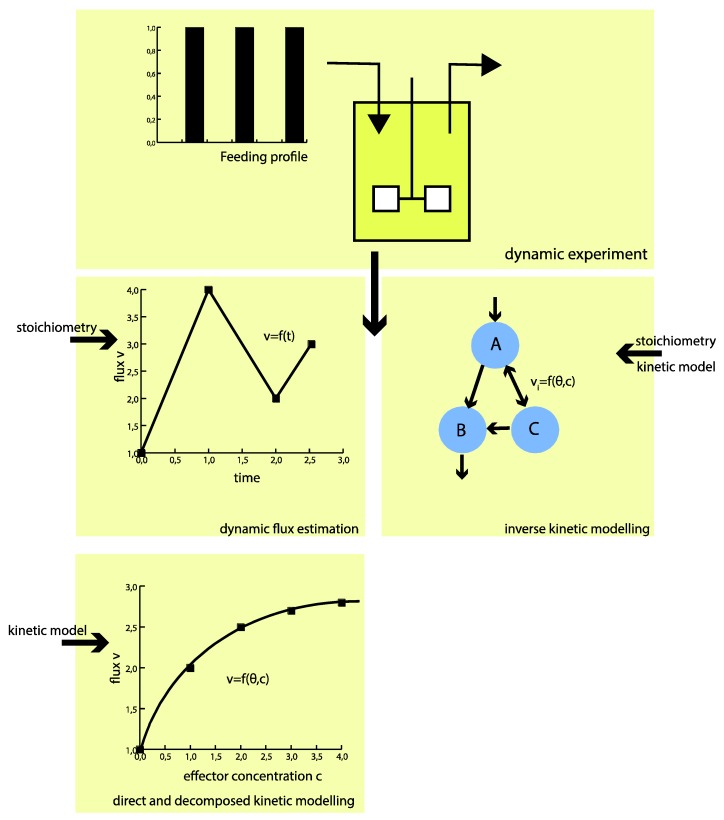
“Classical” kinetic modeling requires *a priori* defined kinetic mechanisms. Using the hybrid modeling approach, only the metabolic network structure (incl. atom transitions) is required *a priori*. As a result, the flux profile in time can be identified rather than kinetic parameters. With the flux functions on hand, the kinetics of the metabolic network can be investigated decoupled from the overall network.

To circumvent these challenges, the proposed hybrid modeling approach strives for direct identification of the intracellular fluxes without *a priori* assumptions of kinetics (see Figure 1). The approach builds on piecewise affine (PWA) flux functions, which approximate the real fluxes throughout the metabolic network. This means only (more readily available) information on the metabolic network structure, including the atom transitions of each reaction, together with the directionality of the fluxes, is required *a priori*.

The approach, as also presented previously [16,17], could be regarded as an extension of DMFA framework [18], which also builds on PWA flux functions but is only suitable for determined or redundant metabolic networks and for underdetermined systems by incorporation of ^13^C labelling [19]. Therewith the resulting parameter estimation problem becomes much more challenging, as the balances for the tracer atoms introduce non-linearity into the so far linear system, which moreover has to be solved by numerical integration instead of analytically. The metabolic tracers add a second set of observables, leading to a multi-objective parameter estimation problem that requires definition of a trade-off between the different types of observables. Moreover parameter correlations, and especially high dimensionality, need to be tackled. Classical non-linear optimization approaches were not found practical for the arising ill-posed inverse problems at a larger scale as they usually lead to very slow convergence and are often not robust enough for practical application in larger-scale metabolic networks.

### Experimental Requirements for *in Vivo* Dynamic Flux Estimation

Crucial for every modeling approach is sufficient information for the identification of the model parameters from the observables [20]. The identifiability of *in vivo* flux functions strongly depends on the specific metabolic network, the experimental design and the captured observables. This has been extensively analyzed for steady-state [21] and the same methodologies can be used in dynamic conditions .Although the experimental design is not the focus of this contribution, the main challenges and requirements are shortly discussed:

• A stimulus-response experiment should lead to perturbation(s) strong enough to cover a significant (metabolite) concentration space for good identification of the kinetic parameters [22].
• The metabolomics should preferably have a complete coverage of the regarded intracellular metabolic response, e.g., intracellular concentrations, which moreover have to be quantitative.
• Use of a ^13^C labeled substrate for improved flux tracing, as the concentration information alone is mostly not sufficient to identify all intracellular fluxes [18,23].

These experimental requirements are partly conflicting, especially when concentration measurements are performed using ^13^C labeled internal standards [24], as they require non-labeled intracellular metabolite pools for quantification. The putative experimental setups therefore either require repetitive (cyclic) conditions or multiple experimental runs to capture the metabolite and ^13^C enrichment information.

In this work, we build on one dynamic setup that has been demonstrated to be advantageous in facilitating concentration and labeling measurements—there cyclic and block-wise feeding are used leading to so-called feast-famine conditions [25,26]. Compared to other experimental stimulus-response setups, e.g., pulse experiments or step changes, the feast-famine conditions show the following characteristics:

(1) They generate repetitive concentration patterns in time, allowing for dense sampling from multiple cycles as well as application of ^13^C labeling from a single experiment.
(2) The feast/famine perturbation includes both: the transient from limitation to excess as well as a return to limitation in a short timeframe of minutes.
(3) In this setup, the starting metabolite concentrations of each cycle are the same as the endpoint, which means there is no net metabolite accumulation during one cycle (material is washed out with the biomass).

These properties are beneficial for flux identification, as the concentration and enrichment information can be captured over several cycles, *i.e.*, improving data density and accuracy.

## 2. Results and Discussion

### 2.1. Computation of an Initial Set of Breakpoints Using the Concentration Measurements

In the piecewise affine flux estimation approach, the choice of the number and positions of the breakpoints is crucial for a successful approximation of the flux profile in time and they directly determine the achievable convergence of the flux approximation towards the real fluxes as well as the number of parameters to be identified from the observables.

The method requires that the breakpoints for all flux functions in the metabolic network have the same set of breakpoints. To best capture the different response of all the metabolites and fluxes a satisfying compromise of breakpoints has to be found. Changes in the (net) in- and out-fluxes of a metabolite pool influence the metabolite concentration and it is assumed that the major changes in metabolic (net) fluxes are reflected if the flux approximation can reproduce the concentration profile well.

With this assumption, an optimization for the placement of breakpoints (number and times) can be performed using the less complex linear metabolite balances and the concentration observables leaving the ^13^C labeling aside. If the reproduction of enrichment measurement is not satisfactory, additional domains can be introduced at any time in the optimization workflow. Moreover, a trade-off between the number of parameters to be estimated and the convergence that can be achieved with the PWA flux functions has to be found. Especially, a too high number of breakpoints can significantly increase the tendency of the system towards overfitting.

Using *in silico* data, the properties of the domain selection are studied. A small reaction network, further referred to as the spiral model, is simulated under feast-famine conditions and the approximation is compared to the noise-free measurements of the model with respect to:

(1) The number of breakpoints and
(2) The placement of breakpoints.

**Figure 2:**
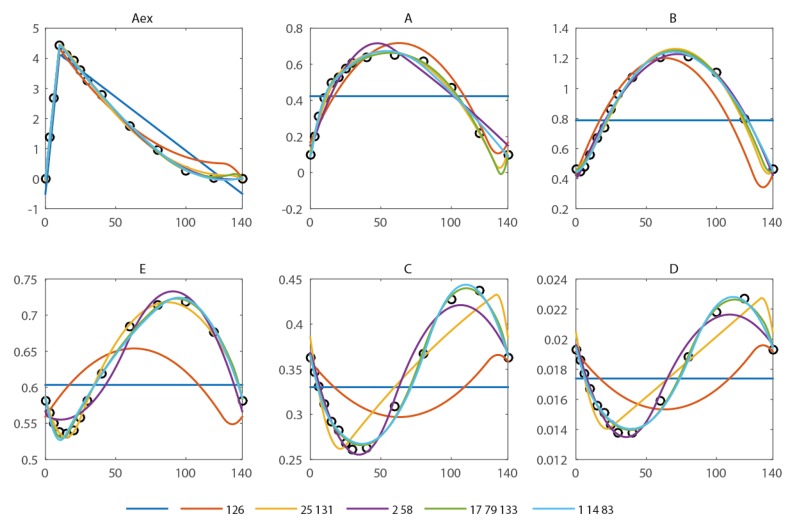
Approximation of the concentration measurements using piecewise affine derivatives with 0 (blue), 1 (orange), 2 (yellow and magenta) and 3 (green and light blue) free breakpoints. The respective residual sum of squared errors can be found in Table 1.

In the spiral network, six metabolite pools are observed (extracellular: Aex, intracellular A, B, C, D, E) at 13 different time points during a feast/famine cycle of 140 s. The first and last breakpoints are fixed (0 and 140 s, the start and end of the feast-famine cycle). With only these breakpoints, no reasonable approximation can be obtained (see Figure 2) and the residual sum of squares is high (579.7). Introducing one additional breakpoint (best at *t* = 126 s) reduces the sum of squares about fourfold (134.1). Nevertheless, the approximation of the concentration profiles is visually not yet sufficient. To compare the goodness of fit we use the *R*^2^ and adjusted ${\bar{R}}^{2}$ with reference to the scenario without any free breakpoint (thus only the fixed points *t*_0_ = 0 s and *t_end_* = 140 s). Further, the Akaike information criterion (AIC) is calculated and summarized in Table 1. All criteria equally suggest a not yet sufficient reproduction (*R*^2^ = 0.77).

**Table 1:** Comparison of goodness of fit for different number of breakpoints and placement. In all cases 98 observations (measurements) are present, the number of parameters corresponds to n × p (n = number of fluxes, p = number of breakpoints).

Break Points (s)	#p	RSS	*R*^2^	${\bar{R}}^{2}$	AIC
-	8	579.7	-	-	91.7
126	16	134.6	0.77	0.72	45.5
25, 131	24	15.1	0.97	0.97	−31.6
2, 58	24	22.2	0.96	0.95	−15.2
17, 79, 133	32	2.3	1.00	0.99	−95.7
1, 14, 83	32	4.7	0.99	0.99	−65.5

With a second free breakpoint (best choice *t*_2_ = 25 s, *t*_3_ = 131 s), a sum of squares of 15.1 (*R*^2^ = 0.97) is reached and the concentration measurements can be reproduced within the expected error range of 10%. Addition of another, third free breakpoint further reduces the RSS to 2.3 (*R*^2^ = 0.99). Next to these global optima, local minima are observed.

When two free breakpoints are placed, the best combination was obtained with *t*_2_ = 23 s and *t*_3_ = 132 s (RSS = 16.9). The territory of approximation error (RSS) has several local minima (data not shown). The second best combination of breakpoints was obtained with *t*_2_ = 2 s and *t*_3_ = 30 s (RSS 25.3).

For three breakpoints, two clusters with low residual are obtained. The global minimum (RSS = 2.3, *R*^2^ = 0.996) is found for a combination with **t** = (17 s, 79 s, 133 s), the second cluster has a minimum of 4.6 at *t*_2_ = 2 s, *t*_3_ = 14 s, *t*_4_ = 83 s (Table 1. Comparison of goodness of fit for different number of breakpoints and placement. In all cases 98 observations (measurements) are present, the number of parameters corresponds to n × p (n = number of fluxes, p = number of breakpoints). These local minima differ in sum of squares about two-fold; nevertheless, based on expected experimental noise both should be taken into account and compared when labeling data is incorporated (isotopomer simulation).

Clearly, the breakpoint optimization will lead to an optimal sequence of breakpoints for the flux functions in terms of the chosen objective function R_c_, but likely never the optimal one, subject to the complete set of observables. Still, the domain selection approach often gives a good sequence of breakpoints in practice. As seen, the implementation of the domain selection in the flux estimation leads to a highly non-convex optimization landscape, and this makes incorporation of the domain selection into the main optimization (using the enrichment information) laborious and computationally hardly feasible on standard computer hardware. From a practical standpoint, it is desirable to start with a simple model, as extension of a model is usually more straightforward than a model reduction, moreover, a smaller number of parameters can often be better identified from the observables [20].

Here the use of objective functions penalizing the number of parameters, like adjusted R^2^ or AIC, can help in finding a minimal set of breakpoints. However, also using those criteria does not necessarily guarantee a sufficient representation of the concentrations, *i.e.*, occurrence of overfitting and no negative concentrations. Having in mind those practical limitations of the domain selection, we implemented constraints in the approach that allow computing flux functions, even with a suboptimal choice of breakpoints and shape-prescriptive constraints.

### 2.2. Introduction of Shape-Prescriptive Constraints

**Figure 3:**
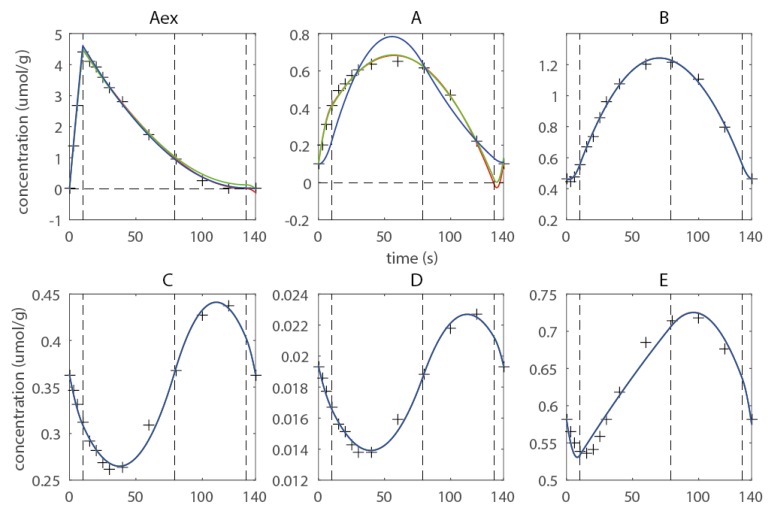
Estimated concentration profile using three free breakpoints **t** = (0s 10s 79s 133s 140s) (**red**), non-negativity constraints (**green**) and shape constraints for metabolite A (**blue**), the corresponding *R_c_* are 4.06 (**red**) 4.60 (**green**) and 72.79 (**blue**).

As an example the sequence of knots **t** = (10 s, 79 s, 133 s) is discussed. Two major flaws can be observed (Figure 3): (1) Negative concentrations have been computed for metabolites A_ex_ and A, and(2) Metabolite A shows overfitting in the last domain. Negative concentrations are undoubtedly not feasible and cannot be accepted; moreover they compromise the numerical integration of the isotopomer balances being detrimental to the numerical robustness of the forward simulation. To enforce non-negativity quadratic inequality, constraints are introduced. This measure prevented all metabolites to reach negative concentrations (Figure 3, green approximation), but does not improve the overfitting behavior observed for metabolite A. Therefore, a shape constraint was introduced enforcing the concentration in the last domain to be monotonous decreasing. The constraint eliminates the overfitting, but leads to higher deviations from the observables in the first and second domain, whereas the fit on A_ex_ is also improved in the last domain with respect to overfitting. Thus, the example illustrates that the introduction of additional constraints is essential as it ensures a feasible concentration profile for the subsequent ^13^C-based estimation of fluxes (see Figure 3). Moreover shape constraints are a very efficient measure to prevent overfitting, and this can also help to smoothen noise in the optimization landscape which leads to a better-posed optimization problem. Moreover they can help to achieve a higher convergence of the flux functions towards the real fluxes, as overfitting in the concentration space can be efficiently eliminated. Considering that the flux estimation problem is usually high-dimensional, the constraints also reduce the parameter space that has to be searched, which can lead to an increase in convergence speed. This example was kept simple for demonstration, an extensively constrained network using experimental data can be found in the next subchapter (also refer to Supporting Material for more details on the introduced constraints).

### 2.3. Estimating Flux Functions Using the Implicit Filtering Algorithm

To demonstrate the practical performance of the optimization approach, a previously described dynamic labeling experiment with *P. chrysogenum* was re-evaluated. The metabolic network for flux identification, further referred to as PenG model, comprises 17 balanced metabolites and 28 fluxes. Five free breakpoints were determined, leading to a total of seven breakpoints, *i.e.*, **t** = (0 s 18 s 36 s 90 s 185 s 230.5 s 360 s). Because of the dependency of the last and first domain (feast/famine setup), 28 × 6 = 168 flux values need to be estimated.

The simulations have been performed over three consecutive feast/famine cycles. As a starting point for the parameter optimization, a feasible parameter set derived from the best fit for the metabolite concentration measurements (including the respective constraints) was used.

**Figure 4:**
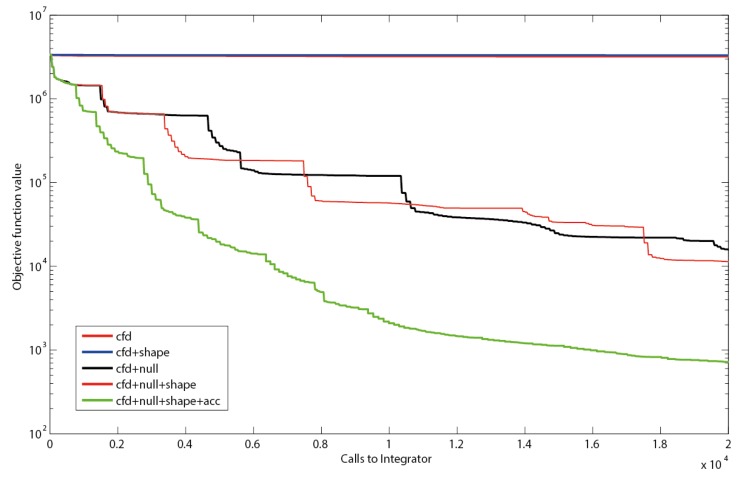
Objective function as a function of executed integrations. Using only central finite differences (cfd), resembling the search directions of a quasi-Newton solver or cfd together with shape constraints (shape) leads to a slow decrease in the residual sum of squares. The use of null-space-based sampling (null) significantly increases the convergence speed. Combining the null-space-based sampling with shape constraints leads to a slight improvement. Even better results are obtained when thresholds for improvement within the current stencil are introduced. Using a threshold of minimally 1% improvement in objective function (acc) to keep the stencil reduces inefficient iterations (green line).

We first applied a damped Quasi Newton optimization algorithm as reference convergence performance (Figure 4). We found a very slow convergence which could originate from (1) stringent hidden constraints or (2) parameter correlations. The slow performance was likely not originating from putative over-stringent constraints, as the speed was even reduced when no constraints were applied. The slow convergence suggests that the PWA optimization problem itself is ill-posed and gradient- based solvers underperform. One reason for the failure could be high correlation of the parameters, which can be taken as an advantage when the correlation is identified and used to determine search directions as implemented in the null-space-based sampling approach.

Null-space-based sampling indeed significantly increases the convergence (Figure 4) and shape constraints even increase the speed further. This increase supports the hypothesis that the shape constraints in the example were not overly stringent nor compromised the optimization landscape for the optimizer, although they have been implemented as hidden constraints in the implicit filtering algorithm. This means for the practical application that all PWA domains can be constrained without loss in performance as long as the constraints are not overly stringent.

It can be observed that the implicit filtering algorithm leads to a step-wise convergence (Figure 4). This behavior can be explained by the stencil rescaling when no parameter set leading to a better value in the objective function is sampled on a particular stencil. Steep descents are observed right after a shrinking of the stencil. After a steep decent a plateau is reached as the stencil is sampled, until no decent direction can be identified anymore, *i.e.*, the gradients become very small. This inefficiency could be reduced by the implementation of a threshold for the improvement between subsequent iterations Indeed, this measure (further referred to as accelerator; acc) improves the convergence significantly, especially for the first couple of iterations. However, it has to be noted that the thresholds can increase the risk of obtaining a local optimum. The strategy that has been implemented to prevent such behavior is to restart the complete sequence of stencil scales after failure of a stencil.

## 3. Materials and Methods

The flux estimation follows a workflow, which will be described in detail in the following subchapters. First step is the domain selection procedure selecting the breakpoints for the flux functions. This is followed by definition and introduction of the non-negativity (prevents negative concentrations) and shape-prescriptive constraints. For use of the final step, first a feasible initial iterate has to be computed as implicit filtering requires this as an input. The final step performs the actual flux identification with incorporation of the enrichment data.

### 3.1. Used Models and Data

The *in silico* Spiral model was used as described previously [16].

For the practical PenG example, concentration and labeling measurements as well as the metabolic network stoichiometry were obtained from a feast/famine cultivation of P. chrysogenum as previously reported [17]. In brief: The culture was supplied by a block-wise feeding regime (36 s feed, 324 s no feed) at an average dilution rate of D = 0.05 h^−1^. The feed contained minimal medium with a glucose concentration of 15 g/L. After several cycles, repetitive offgas and DO measurements were obtained, a biomass concentration of 5.7 g/L was obtained (average over the cycle). Samples for intracellular metabolites were withdrawn using a rapid-sampling device [27], quenched, extracted and analyzed using the ID-MS protocol [17,28]. After sampling for intracellular concentrations, the feed was switched to a medium with the same composition, but containing fully ^13^C-labeled glucose. The enrichment was monitored during three consecutive cycles using rapid sampling and MS analysis.

### 3.2. Mathematical Modeling of Dynamic ^13^C Labeling Experiments Using PWA Flux Functions

The modeling approach is based on balances for metabolite concentrations and enrichments quantified by isotopomers (cumumers or other formalisms) [23,29]. For isotopically non-stationary labeling the enrichment can also be simplified using C-molar enrichment (average enrichment of a metabolite over all C-atoms) [17]. The respective balances are generated based on the stoichiometry of each reaction and the respective C atom transitions. As the approach is well documented in literature (especially for steady-state), only an abstract description is given. The metabolite balance is based on the flux functions α and the metabolic network stoichiometry **N**.

(2) $$\frac{dc}{dt}=f(N,\alpha )$$

Based on the atom transitions and the stoichiometry, a non-linear function *g* can be derived describing the balance of each single isotopomer concentration (or cumumer, EMU, *etc.*) *x* of the network:

(3) $$\frac{dx}{dt}=g(c,\alpha ,x,x^{inp})$$

Integration of the isotopomer balances will yield the time-course of the isotopomer concentrations, which can be used to calculate the observables, *i.e.*, mass-isotopomer fractions.

### 3.3. Flux Functions and Nomenclature in the PWA Flux Framework

Using piece-wise affine functions for the flux description changes some properties of the equation system. The piece-wise affine flux functions are defined in time by a set of breakpoints t_j_ valid for all fluxes in the considered metabolic network, partitioning the flux function v_i_ into number of breakpoints minus one domains in time:

(4) $$v_{i,j}\left(t\right)=\{\begin{array}{cc} v_{i,0}+\frac{v_{i,1}-v_{i,0}}{t_{i,1}}t & t_{j=0}\leq t\leq t_{1}\\ v_{i,1}+\frac{v_{i,2}-v_{i,1}}{t_{i,2}-t_{i,1}}\left(t-t_{1}\right) & t_{1}<t\leq t_{2}\\ v_{i,2}+\frac{v_{i,3}-v_{i,2}}{t_{i,3}-t_{i,2}}\left(t-t_{2}\right) & t_{2}<t\leq t_{3} \end{array}$$

The parameters of this function are the value of respective fluxes v_i_ at the breakpoint t_j_ and defined strictly positive (so a reversible reaction will have two fluxes). Flux values between breakpoints are calculated by linear interpolation on its two adjacent breakpoints. Therefore a metabolic network with i fluxes and j breakpoints has i × j parameters to be estimated. As will be discussed later, this number can decrease for specific experimental setups that introduce additional constraints on the concentrations or flux functions. This definition is also valid for higher order piecewise-defined flux functions as long as they are to be defined unique on the breakpoints, e.g., smooth quadratic splines, but will not be discussed in the scope of this paper.

### 3.4. Balancing of Metabolites and Solution of the Metabolite Mass Balances

With the previous definition of the flux functions in time, the metabolite mass balances can be formulated locally at every breakpoint *j* by linear combinations according to the stoichiometry matrix which can be further divided into known *v_b_* and unknown *v_n_* fluxes [30].

(5) $$\begin{array}{l} N=[N_{b}\;N_{n}]\\ \frac{dc}{dt}(t_{j})=N_{b}v_{b}+\;N_{n}v_{n}\\ v_{n}={N_{n}}^{-1}\left(\frac{dc}{dt}(t_{j})-N_{b}v_{b}\right) \end{array}$$

If N_n_ is square and nondegenerate, the matrix can be inverted and a unique solution of unknown fluxes can be identified from the concentration transients and the known fluxes.

(6) $$v_{n}={N_{n}}^{-1}\left(\frac{dc}{dt}(t_{j})-N_{b}v_{b}\right)$$

In practice most metabolic networks are underdetermined with respect to the number of unknown fluxes f, *i.e.*, they contain parallel pathways, cycles or reversible reactions.

(7) $$rank(N_{n})<f$$

This means the metabolic network does not contain enough measurable (known) fluxes in order to identify all fluxes. In this case Nn spans a null space (kernel), which contains all linear combinations of its basis vector(s) describing the infinite combinations of fluxes that lead to the same solution of $\frac{dc}{dt}$.

(8) $$\left\{v_{n}+w|N_{n}\;v_{n}=\text{const}\wedge w\in Null(N_{n})\right\}$$

This is called practical non-identifiability and also directly explains why isotopic tracers are needed in order to identify all flux functions of such metabolic networks.

The right hand side (rhs) $\frac{dc}{dt}$ is a linear combination of the flux functions **v**, thus $\frac{dc}{dt}$ also results in a PWA function. Therewith the rhs can be described by a linear function **u** on the same breakpoints as **v**:

(9) $$\frac{dc}{dt}(t)=u(t)\;\;\;\;\;\;\text{with}:\;\;\;u\left(t\right)=\{\begin{array}{cc} u_{0}+\frac{u_{1}-u_{0}}{t_{1}}t & t\leq t_{1}\\ u_{1}+\frac{u_{2}-u_{1}}{t_{2}-t_{1}}\left(t-t_{1}\right) & t_{1}<t\leq t_{2}\\ u_{2}+\frac{u_{3}-u_{2}}{t_{3}-t_{2}}\left(t-t_{2}\right) & t_{2}<t\leq t_{3} \end{array}$$

This function can easily be integrated (analytical), leading to a continuous, quadratic solution for the concentration in time with the initial concentrations **c**_0_:

(10) $$c(u,t)=c_{0}+\{\begin{array}{ll} u_{0}\;t+\frac{1}{2}\frac{u_{1}-u_{0}}{t_{1}}\;\;t^{2} & t\leq t_{1}\\ u_{0}\;t_{1}+\frac{1}{2}\left(u_{1}-u_{0}\right)\;t_{1}+u_{1}\;\left(t-t_{1}\right)+\frac{1}{2}\frac{u_{2}-u_{1}}{t_{2}-t_{1}}\;{\left(t-t_{1}\right)}^{2} & t_{1}<t\leq t_{2}\\ u_{0}\;t_{1}+\frac{1}{2}\left(u_{1}-u_{0}\right)\;t_{1}+u_{1}\left(t_{2}-t_{1}\right)+\frac{1}{2}\left(u_{2}-u_{1}\right)\;\left(t_{2}-t_{1}\right)+\left(u_{3}-u_{2}\right)\left(t-t_{2}\right)+\frac{1}{2}\frac{u_{3}-u_{2}}{t_{3}-t_{2}}{\left(t-t_{2}\right)}^{2} & t_{2}<t\leq t_{3}\\ \vdots & \vdots \end{array}$$

As can be seen, this solution is moreover linear with respect to *u_i,j_*; with available concentration measurements, a linear regression can be performed to obtain the best estimate of **u**. The measurements **c**_M_ at timepoints **t**_M_ can be obtained by generation of a matrix **Y**:

(11) $$c_{M}=\underset{=Y}{\underset{︸}{\left(1^{T}\;\;\;Y^{*}\right)}}\;\left(\begin{array}{c} c_{0}\\ u \end{array}\right)$$

The weighted linear regression problem (with weight matrix **W**_M_) then reads:

(12) $$\left(\begin{array}{c} c_{0}\\ u \end{array}\right)={\left(Y^{T}\;W_{M}\;Y\right)}^{-1}Y^{T}\;W_{M}\;Y\;c_{M}$$

Solving the optimization problem

(13) $$\hat{\alpha }=\text{arg}\underset{\alpha }{\text{min}}R_{c}$$

### 3.5. Sufficient Breakpoint Selection

**Figure 5:**
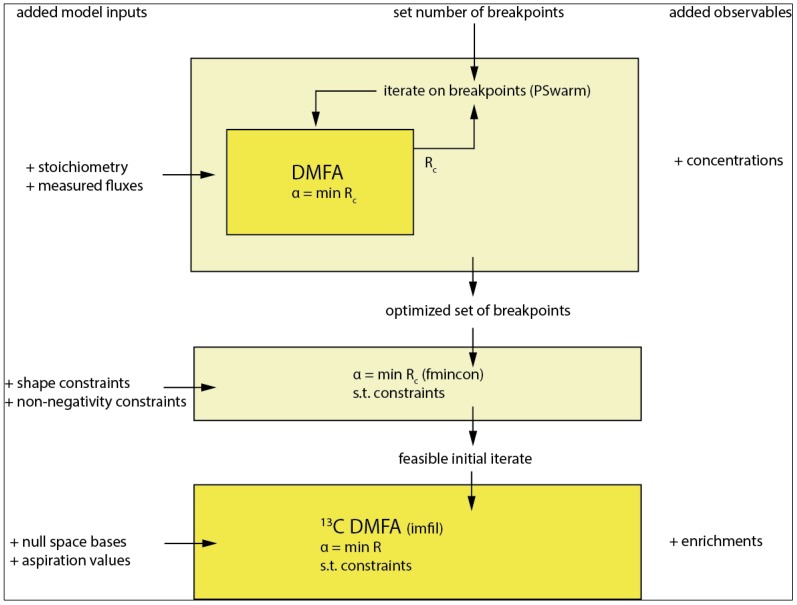
Overall workflow of the method with breakpoint selection as the first step followed by the main optimization and used optimizers in parentheses. The model inputs are successively expanded as described on the left columns. The first two steps only use the concentration observables, whereas the final step additionally incorporates the enrichment observables from the ^13^C tracer.

For the flux functions the location of the breakpoints in time has to be selected as also derived in [18]. Best practice would be to use all observables (concentration and enrichments) for this selection; however, considering the complexity and dimensionality of the resulting parameter optimization problem, this is computationally not feasible on standard computer hardware. The optimization is implemented in a nested manner (see Figure 5. Overall workflow of the method with breakpoint selection as the first step followed by the main optimization and used optimizers in parentheses. The model inputs are successively expanded as described on the left columns. The first 2 steps only use the concentration observables whereas the final step additionally incorporates the enrichment observables from the ^13^C tracer.) with the inner loop solving Equation (12). and the outer loop iterating on the breakpoints minimizing the weighted residual sum of squares on the concentration observables R_c_ using the *PSwarm* optimization algorithm [31] (Figure 5).

(14) $${\hat{t}}_{j}=\text{arg}\;\underset{t_{j}}{\text{min}}\;R_{c}$$

From *R_c_* the adjusted *R*^2^ as well as the AIC have been calculated [32,33].

### 3.6. Introducing Constraints

In practical applications, measurement data with random errors (noise) can lead to issues during the optimization. Especially, cases that impair the stability of the numerical integration have to be prevented to ensure maximal robustness of the high-dimensional parameter estimation: (1) Negative values in the approximation of the concentration profile; (2) Overfitting. Additionally, the experimental setup could enforce certain conditions that should be reflected in the parameter estimation, like in the case of the feast/famine setup, the first and last domain (beginning and end of the cycle) have to be equal.

#### 3.6.1. Specific Constraints for Feast-Famine Conditions

In the previous paragraph, the solution for the best estimate of the concentration profile with the given breakpoints has been demonstrated. In the special case of a feast/famine experiment the solution should additionally fulfill two (linear) constraints:

(1) In a stable feast-famine regime the metabolite concentration at the end of one cycle has the same concentration as in the beginning (of the next cycle).
(2) Similarly, the flux at the end of the feast famine cycle has to be the same as in the beginning. Otherwise no stable, repetitive cycles were obtained.

The concentration at the end of the cycle is the result of the integration using the parameters **u** (and *c*_0_). A column vector **C** is generated, representing a column of matrix **Y** for the timepoint *t_end_*:

(15) $$C=Y_{t_{end}}$$

Integration of the constraint is obtained using Lagrange multipliers λ:

(16) $$\left(\begin{array}{c} \hat{q}\\ \lambda \end{array}\right)=Z\left(\begin{array}{c} Y^{T}W_{M}Yc_{M}-c_{0}\\ b \end{array}\right)\;with\;Z=\left(\begin{array}{cc} Y^{T}W_{M}Y & C^{T}\\ C & 0 \end{array}\right),\hat{q}=\left(\begin{array}{c} u\\ {\hat{c}}_{0} \end{array}\right)$$

A comparable approach is taken to introduce the flux constraint *v*_1_ = *v*_j_. The vector **C** (and **b**) are extended by an additional row:

(17) $$C=\left(\begin{array}{cccc} 1 & 0 & \ldots & -1 \end{array}\right),\;\;\;b=0$$

As can be seen this equality constraint reduces the number of parameters by one per balanced metabolite.

#### 3.6.2. Non-Negativity Constraints

In order to ensure numerical stability of the integration for the isotopomer equations, it is crucial that computed negative approximated concentrations are detected and eliminated. In order to find a sufficient constraint the piecewise quadratic concentration pattern has to be evaluated in every domain. In the convex case, the minimum of the vertex value has been computed, whereas in the concave case it has to be checked for roots in its interval. These computations are trivial but correspond to a nonlinear (quadratic) inequality constraint.

#### 3.6.3. General Shape-Prescriptive Constraints

In practical applications overfitting has to be prevented. Reasons for overfitting can be (1) noisy data, (2) outlier or leverage points, (3) suboptimal choice of domains for flux functions, (4) suboptimal sample time points, (5) systematic error in observables or (6) missing data. Overfitting will lead to flux functions with potentially too high gradients. Additionally, because metabolite pools are often closely linked, the overfitting will propagate through the metabolic network, leading to an ill-posed optimization landscape hindering the parameter estimation progression and biased flux functions.

One obvious approach is to optimize the domain selection as discussed. Nevertheless, there are limits to the reduction of domains as all flux functions are defined on the same domains, and a trade-off between overfitting prevention and achievable convergence of the flux functions has to be found. It has to be noted that the proposed linear solution of the domain selection can only be applied to a subset of the observables (the concentration measurements), while non-linear differential equation systems are required to obtain the labeling profile. Consequently, the domains are chosen on a subset of the available data leading to a risk that the domain selection yields a good approximation but potentially not the global optimum for all data.

Shape constraints can be applied to “guide” the optimization procedure using shape primitives; together with the least squares objective function. This additional information on the shape of the (quadratic) concentration patterns has several benefits:

(1) It can facilitate higher convergence orders of the flux functions; (2) The constraints restrict the search space and prevent that the optimization algorithm to converge in local optima in a noisy optimization landscape; (3) The chosen constraints do not introduce additional (unobserved) parameters; (4) The evaluation of the constraints is computationally very cheap even at large scale.

Shape constraints are (with exception of the non-negativity constraint) linear inequality equations, which can be implemented rapidly and intuitive in the PWA framework as linear inequality constraints on the parameter vector. This also means that feasible parameter sets can be computed almost instantly using linear programming. It has to be noted that shape constraints can also be introduced for metabolites where no observables are on hand (e.g., the metabolite is very unstable and cannot be quantified) which are exceptionally prone to overfitting as they do not contribute to the objective function. A strategy that has been used in the Penicillium model was to assume non-observed pools to follow the same dynamics as their surrounding metabolites.

#### 3.6.4. Monotonicity and Convexity Constraints

**Figure 6:**
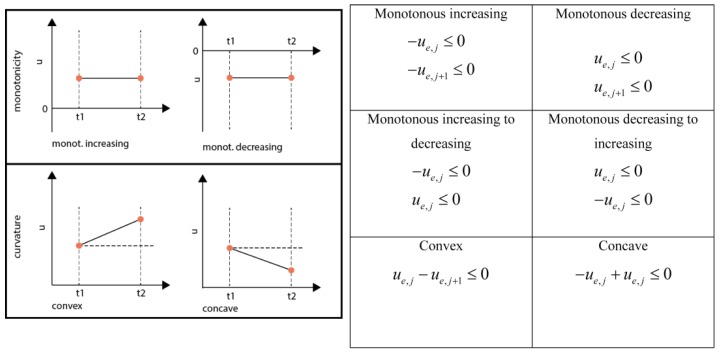
Inequality equations for the different shape constraints.

When constraining the monotonicity in a domain four cases have to be considered for PWA flux functions: (1) Monotonous decreasing, (2) Monotonous increasing, (3) Switch from monotonous increasing to monotonous decreasing and (4) Switch from monotonous decreasing to monotonous increasing (See Figure 6). In the case of a quadratic function this behavior can be enforced by constraining the right hand side (rhs) **u** at the breakpoints adjacent to the respective domain.

A direct switch from monotonous increasing to decreasing and the other way round is only possible if the rhs between those neighboring domains was zero, leading to a double-inequality constraint which is equivalent to an equality constraint, *i.e.*, forcing the inflection point to the respective middle knot. Generally, convexity and curvature can be constrained independently. Nevertheless, in the case that a change of monotonicity is enforced, also the curvature is constrained. It is good practice to check the constraints for feasibility, e.g., by using a linear programming solver, e.g., linprog in MATLAB.

#### 3.6.5. Equality Shape Constraints

In special cases a more stringent constraint than the inequality constraints could be needed. Here, the slope of the concentrations at the breakpoints can also directly be constrained using an equality constraint:

(18) $$u_{e,j}=A$$

This constraint has been applied in the Penicillium model for metabolites with very noisy concentration measurements that did not allow for identifying a trend. The slopes have been set to zero, *i.e.*, the (net) influx equals the outflux at all times (details can be found in the Supplementary Material).

### 3.7. ^13^C DMFA

The final flux (parameter) estimation problem has to minimize the error subject to two sets of objectives: (1) The concentration measurements and (2) The ^13^C enrichment observables. The weighted residual sum of squares (*R*) was chosen, whereas a constant *a* was introduced as scaling factor to weight the two *R_i_*, which will lead to different points on the respective pareto frontier; it has been set to 1.317 to normalize for the different number of observations in either dataset. This leads to a multi-objective optimization problem and the L_2_ global criterion was used to define the overall objective function on previously computed aspiration values (best fit on either dataset) for the two residual sum of squares (*R_c_* and *R_x_*).

(19) $$\begin{array}{ll} R_{c} & =a\cdot \sum_{m}{\left(\frac{y_{c,m}-y_{c,m}\left(\theta ,\text{t}\right)}{\sigma_{c,m}}\right)}^{2},\;\;\;\;\;\;R_{x}=\sum_{m}{\left(\frac{y_{x,m}-y_{x,m}\left(\theta ,\text{t}\right)}{\sigma_{x,m}}\right)}^{2}\\ R & =\left({\left(\frac{R_{c}-R_{c,asp}}{R_{c,asp}}\right)}^{2}+{\left(\frac{R_{x}-R_{x,asp}}{R_{x,asp}}\right)}^{2}\right)\\ \hat{\theta } & =\text{arg}\;\underset{\theta }{\text{min}}\;R\\ & s.t.\\ & shape\;const.\\ & nonneg.\;constr. \end{array}$$

Computing the aspiration values on first sight seems an unnecessary additional effort, but it has to be noted that the best fit of the concentration data is already available from the domain selection procedure and a good approximation of the enrichments can usually be computed fast, neglecting the concentration observables.

In practice this has the advantage that the whole dataset can be inspected for outliers and leverage values and those observations can be removed or downweighted. This procedure will lead to a better posed optimization landscape and can speed up the overall parameter estimation process considerably. If no good estimate for the errors is available, those could also be estimated using, e.g., an iteratively reweighted least squares approach like robust regression. With the current scope to analyze the practical optimization convergence, a simplifying assumption of an error of 5% of the average value within each dataset was used.

#### 3.7.1. The Implicit Filtering (Imfil) Optimization Algorithm

The implicit filtering algorithm has been applied as provided (version for MATLAB) [34] with minor modifications and bug fixes. In brief, implicit filtering is a hybrid sampling algorithm that samples points on a so called *stencil* (with variable size in the parameter space called *scale* centered at the current best iterate) in the parameter space comparable to a pattern search or direct search algorithm. From the collected points in the iteration, projected gradients are computed, which are then used to perform a quasi-Newton iteration. In case no improvement was found using the quasi-Newton iteration, the solver will move the center of the stencil to the best sampled point. If no better point was sampled, the actual stencil scale is adjusted (decreased) or the optimization is terminated. In this work, the quasi-Newton iteration with the SR1 Hessian update was used.

#### 3.7.2. Implementation of the Constraints in Implicit Filtering (Imfil)

**Figure 7:**
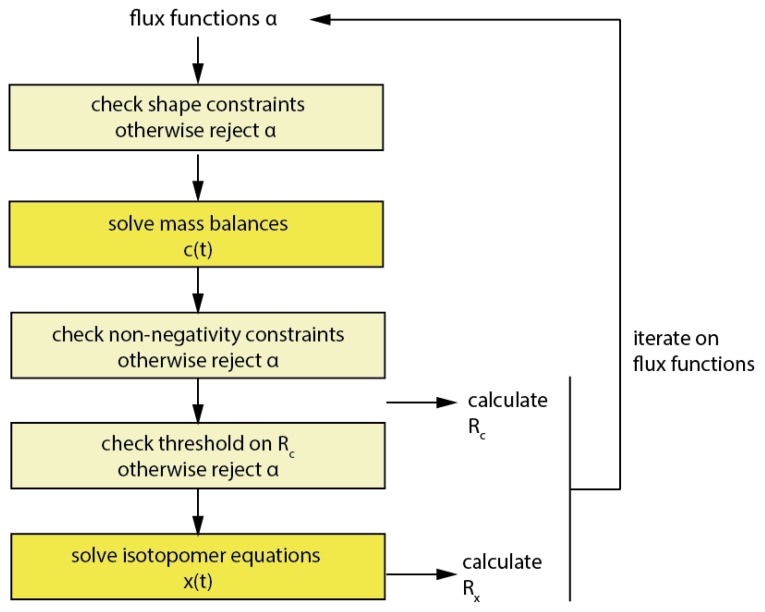
Workflow of the optimization in *imfil*.

*Imfil* has to be initialized with a strictly feasible initial parameter set, which was computed using the MATLAB *fmincon* solver. In contrast to *fmincon*, using *imfil*, constraints have to be implemented as so-called hidden constraints, *i.e.*, a parameter set outside the feasible territory is rejected and a failure is returned to the algorithm (Figure 7).

This might at first seem overly simplistic, but considering that in the code 99.8% of the computational time in the forward simulation is being spent for the numerical integration of the isotopomer equations, rejecting parameter vectors without performing the numerical integration for non-feasible parameter sets can reduce the computational cost notably. Therefore all mentioned constraints have been introduced as hidden constraints when *imfil* was used. Beside the already discussed constraints, also a constraint on R_c_ has been implemented, and this was typically set to two times the initial R_c_ and prevents that a numerical integration of flux functions is performed for parameter vectors with a very bad objective function value on the concentrations. It is also useful in the initial phase of the optimization, when R_x_ dominates the objective function, in order to constrain the redistribution of error into R_c_.

#### 3.7.3. Null-Space-Based Sampling

Hidden constraints can have the risk of compromising the identification of a decent gradient. Therefore, it is beneficial to enrich the stencil with additional directions that (1) are likely to lead to a better value in objective function and (2) are known to fulfill the given constraints together with the central finite differences. Feasible directions have been implemented in the algorithm using the *vstencil* property of the solver.

A powerful set of such directions can be directly derived from Equation (7). Samples within this null space will have the same rhs u, and therewith the concentration profile as the current best iterate, but still a different solution of the isotopomer balances. Using this stoichiometry-based null space is further referred to as null-space-based sampling.

**Figure 8:**
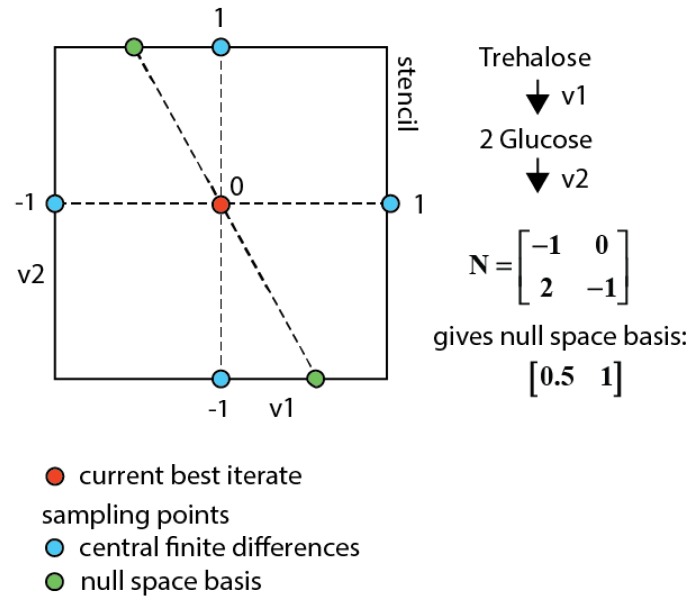
Sampling space for a network with two fluxes and one measured metabolite (glucose). When using central finite differences the blue points are sampled. Using the null space sampling additionally the two green points are sampled that have the same concentration residuals as the current best iterate and therewith also always fulfill the constraints.

This approach allows iterating in the lower dimensional space of fluxes (parameters) (see Figure 8), which cannot be determined by the network stoichiometry and extracellular rates, but requiring ^13^C labeling measurements to be identified. This parameter space reduction also eliminates the putative trade-off between concentration and enrichment observable residuals in the multi-objective function (*i.e.*, the concentration residual remains equal). In order to find the best overall optimum on all observables the central finite differences for all parameters are sampled together with the null space basis vectors.

## 4. Conclusions

Dynamic flux identification requires advanced experimental and computational approaches, especially experimental setups that allow for the simultaneous, respective serial measurement of intracellular concentrations and labeling enrichments. For the evaluation and interpretation of this experimental data, modeling and parameter estimation are crucial and require attention to obtain a good approximation of metabolic network fluxes.

Metabolic networks show an inherent correlation of (flux) parameters and common optimization approaches fail to identify the proper search direction. To solve this issue, we applied PWA flux functions and a two-staged optimization that resulted to be feasible also for larger networks. Especially, the implementation of implicit filtering-based optimization algorithms increased the convergence speed and basically eliminates manual intervention by frequent re-initialization of the optimization. The proposed implementation allowed for the obtaining of a reasonable estimate for 168 parameters within 20,000 function evaluations. Key for fast convergence were (1) implementation of null space sampling and (2) shape constraints that prevent overfitting and reduce the search space.

Once the flux functions in time have been identified, these together with the respective effector concentration measurements allow for testing of different biological hypothesis on the regulatory mechanism. In contrast to the classical reaction kinetic approach, the identification of kinetic parameters can now be decoupled from the overall metabolic network, *i.e.*, each approximated flux profile can be reproduced and tested using different kinetics formats for a small network as discussed in [16].

## Supplementary Materials

Supplementary materials can be accessed at: http://www.mdpi.com/2218-1989/5/4/697/s1.

## Symbols and Abbreviations

Subscript	
0	Initial condition
asp	Aspiration value
e	Metabolite identifier
end	End of feast famine cycle
i	index of flux
j	index of breakpoint
k	index of domain
m	Index of measurement
M	All measurements
Superscript	
inp	Enrichment of used substrate for labelling
b	Known flux
n	Unknown flux
f	Number of unknown fluxes
General	
a	Scaling factor
e	Enzyme activity
R, RSS	(weighted) residual sum of squares
R^2^	Coefficient of determination
θ	Flux function parameters
α	Kinetic parameters
c	(metabolite) concentration
x	(C-molar) enrichment
v	Flux
u	Right-hand-side (dc/dt)
W	Weight matrix
^	Optimal estimate
t	Time
λ	Lagrange multiplier
w	Translation vector of the null space
N	Stoichiometry matrix
σ	Standard deviation
imfil	Implicit filtering algorithm
PWA	Piecewise affine
acc	Accelerator for imfil
DMFA	Dynamic metabolic flux analysis
AIC	Aikaike information criterion
