# Supplementary material for: Effective Estimation of Dynamic Metabolic Fluxes Using 13C Labeling and Piecewise Affine Approximation: From Theory to Practical Applicability
Source: Metabolites. 2015 Dec 4;5(4):697–719. doi: 10.3390/metabo5040697 (PMC4693191; doi:10.3390/metabo5040697)
Supplement: Supplementary file 1 [file metabolites-05-00697-s001.pdf]

OPEN ACCESS
metabolites
ISSN 2218-1989
www.mdpi.com/journal/metabolites/

Supplementary Materials

1. The spiral network

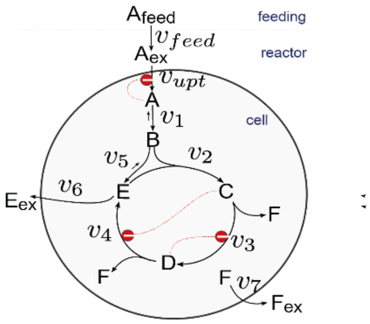

Figure S1. Network stoichiometry of the spiral network.

2. The PenG Network

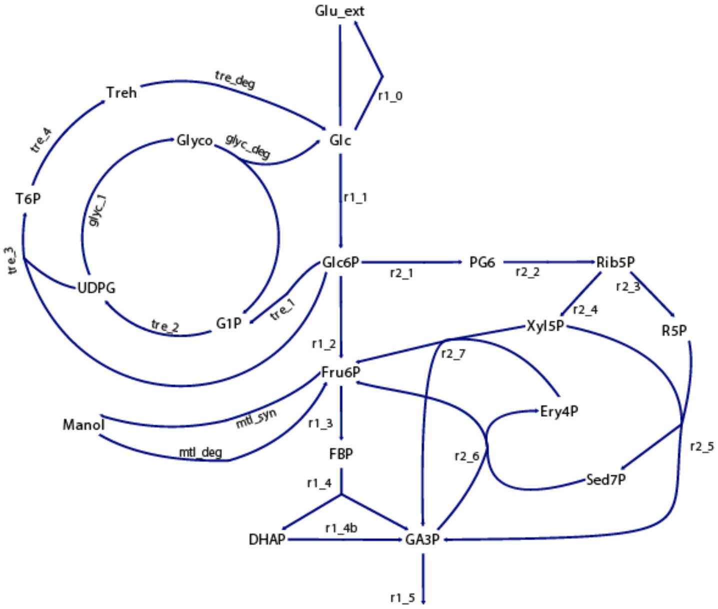

Figure S2. Network stoichiometry of the PenG network.

3. Estimated Flux Functions with Respective Concentration and c-Molar Isotopomer Simulations

It has to be noted that, for simplification, the weights for every series of observables have been set to 5% of the average of the respective series. Those weights are different to the ones of the previously published flux functions. Moreover, the previously published fit did not optimize a multi-objective objective function but constrained all right hand sides at the concentration breakpoints to the values of the best concentration fit and only optimized the enrichments. Therefore, the fits represent different points on the pareto frontier between the two sets of observables.

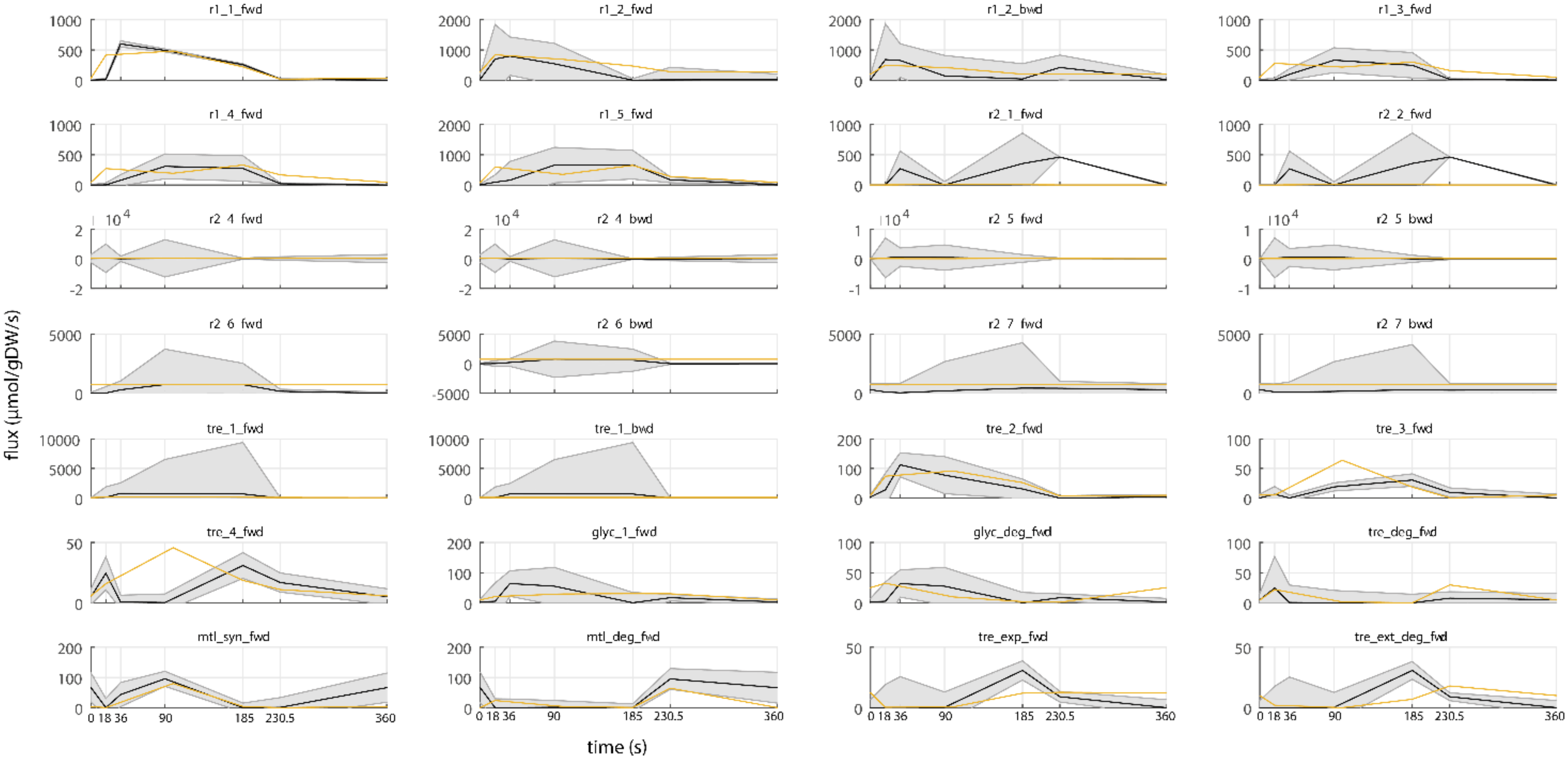

Figure S3. Estimated flux functions with asymptotic confidence intervals in comparison to the previously published best fit.

Table S1. Best flux estimates found for the PenG model, all values in umol/gDW/h.

	0 s	18 s	36 s	90 s	185 s	230.5 s	360 s
**r1_1_fwd**	0.691	11.581	600.000	493.116	257.404	18.137	0.691
**r1_2_fwd**	30.581	698.237	795.750	542.587	1.869	10.618	30.581
**r1_2_bwd**	22.753	682.819	652.364	143.461	40.008	421.277	22.753
**r1_3_fwd**	3.250	1.875	96.850	331.080	246.959	13.957	3.250
**r1_4_fwd**	0.166	1.875	77.915	307.633	274.415	21.002	0.166
**r1_5_fwd**	5.889	92.760	158.672	657.274	671.126	171.393	5.889
**r2_1_fwd**	0.177	0.661	268.952	2.105	354.458	461.993	0.177
**r2_2_fwd**	0.000	0.156	267.510	1.472	355.794	462.311	0.000
**r2_4_fwd**	251.235	451.182	124.624	446.247	251.394	331.438	251.235
**r2_4_bwd**	255.277	454.356	17.191	422.894	2.148	18.469	255.277
**r2_5_fwd**	3.111	352.153	629.308	471.102	175.334	164.837	3.111
**r2_5_bwd**	5.171	348.843	504.904	499.518	52.833	2.090	5.171
**r2_6_fwd**	8.390	23.220	295.129	743.652	736.643	168.366	8.390
**r2_6_bwd**	10.455	29.826	210.018	762.307	603.623	4.500	10.455
**r2_7_fwd**	305.267	114.414	47.250	208.708	436.804	424.321	305.267
**r2_7_bwd**	307.332	121.021	113.033	156.940	301.889	259.870	307.332
**tre_1_fwd**	10.100	143.243	796.548	750.272	701.161	8.956	10.100
**tre_1_bwd**	7.239	116.443	702.885	693.160	683.240	19.389	7.239
**tre_2_fwd**	4.053	28.071	112.468	77.594	30.886	0.109	4.053
**tre_3_fwd**	0.280	6.466	0.230	18.887	30.488	9.515	0.280
**tre_4_fwd**	5.185	24.505	0.691	0.292	30.943	16.851	5.185
**glyc_1_fwd**	2.975	5.332	64.066	55.156	0.398	17.235	2.975
**glyc_deg_fwd**	1.487	2.666	32.033	27.578	0.199	8.618	1.487
**tre_deg_fwd**	5.185	24.505	0.691	0.021	0.095	7.783	5.185
**mtl_syn_fwd**	66.168	0.169	43.371	95.695	0.000	0.911	66.168
**mtl_deg_fwd**	66.168	0.125	0.052	1.127	0.649	95.153	66.168
**tre_exp_fwd**	0.000	0.000	0.000	0.271	30.847	9.069	0.000
**tre_ext_deg_fwd**	0.000	0.000	0.000	0.271	30.847	9.069	0.000

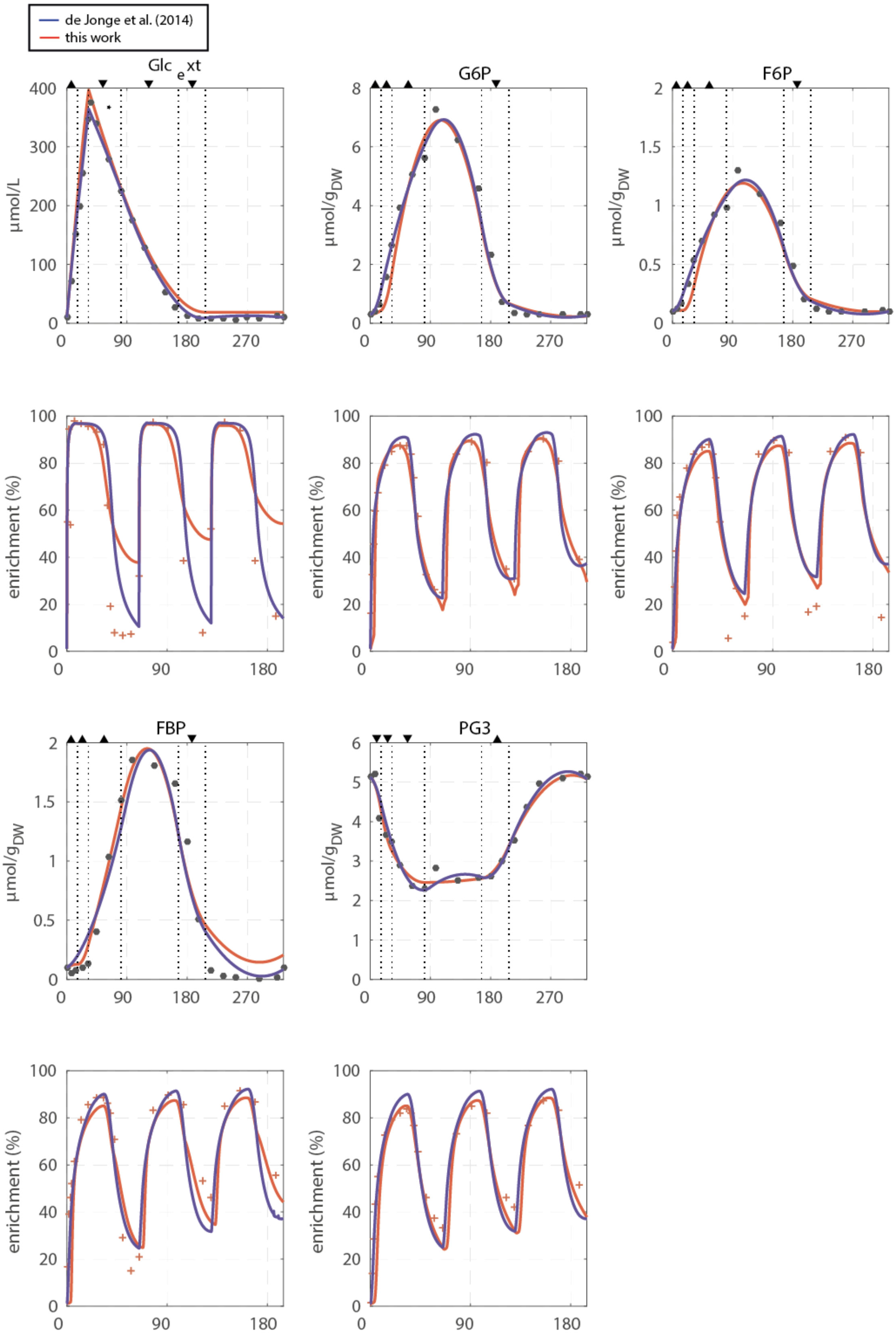

Figure S4. Comparison of previously published (**blue**) and best parameters found using the implicit filtering algorithm. Triangles on the top indicate the introduced shape constraint; monotonous increase (**upwards pointing triangle**) and monotonous decrease (**downwards pointing triangle**), no convexity constraints have been introduced.

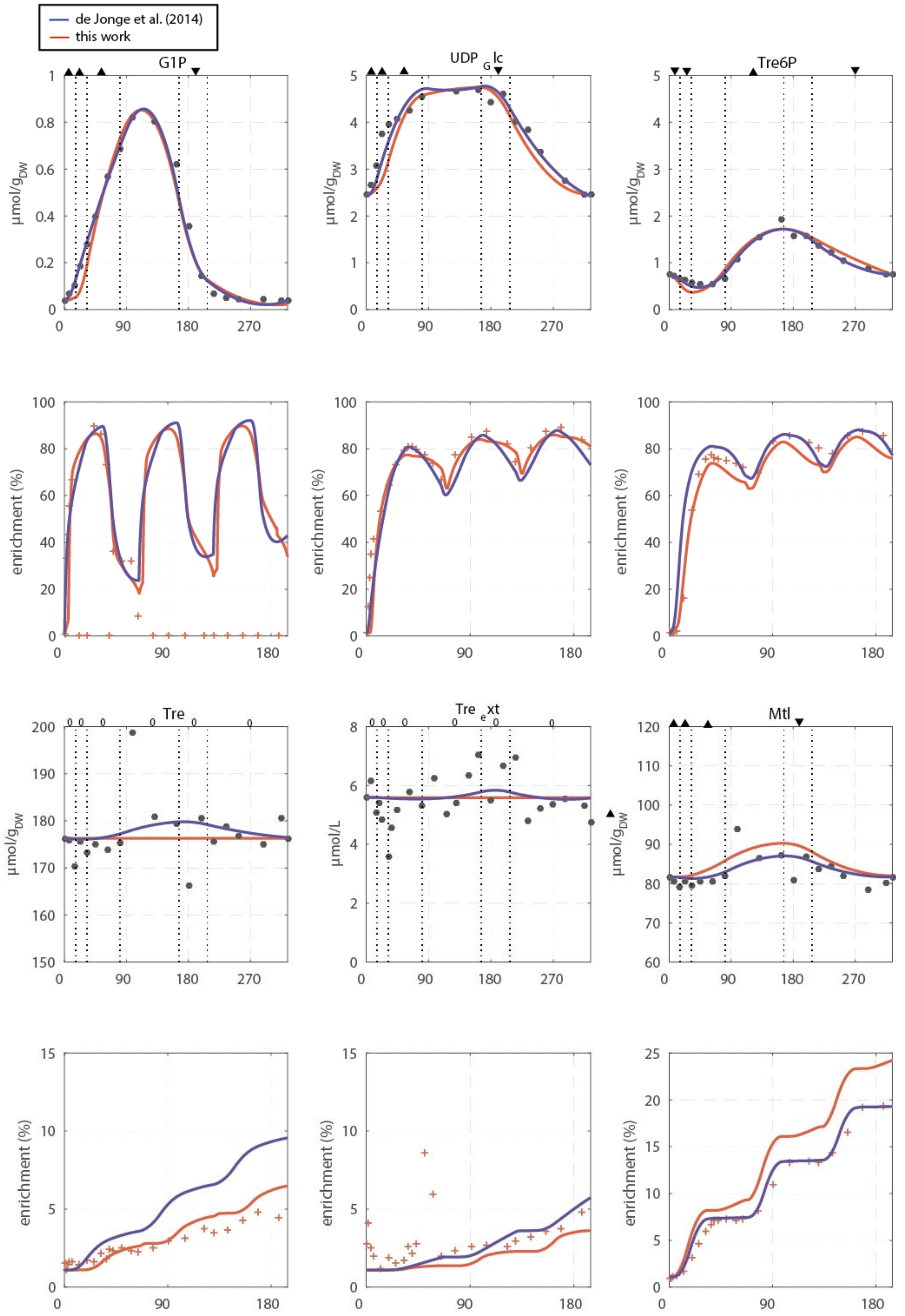

Figure S5. Comparison of previously published (**blue**) and best parameters found using the implicit filtering algorithm. Triangles on the top indicate the introduced shape constraint; monotonous increase (**upwards pointing triangle**) and monotonous decrease (**downwards pointing triangle**), no convexity constraints have been introduced.

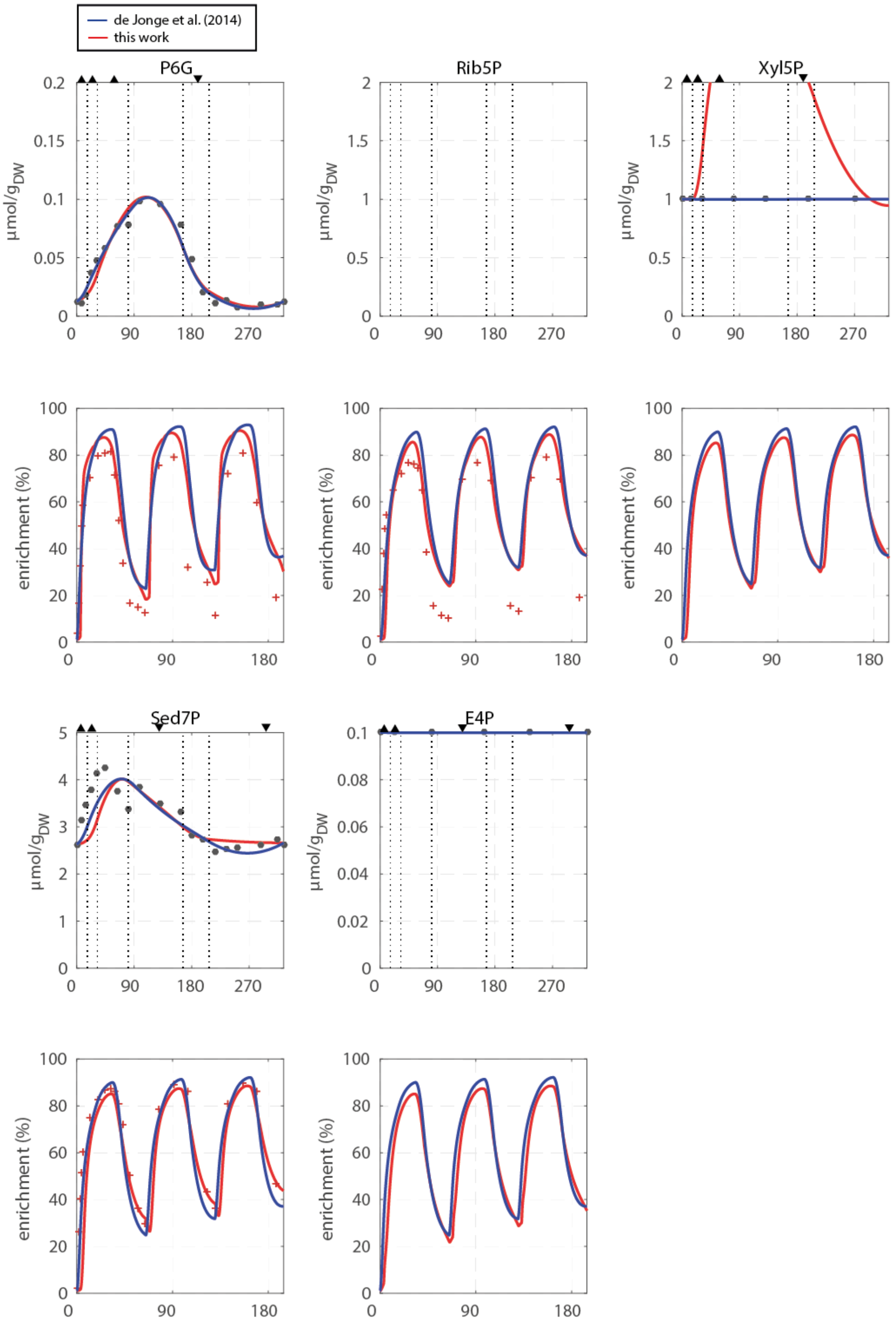

Figure S6. Comparison of previously published (**blue**) and best parameters found using the implicit filtering algorithm. Triangles on the top indicate the introduced shape constraint; monotonous increase (**upwards pointing triangle**) and monotonous decrease (**downwards pointing triangle**), no convexity constraints have been introduced.

© 2015 by the authors; licensee MDPI, Basel, Switzerland. This article is an open access article distributed under the terms and conditions of the Creative Commons Attribution license (http://creativecommons.org/licenses/by/4.0/).
